# Quantitative Evaluation of Rabbit Brain Injury after Cerebral Hemisphere Radiation Exposure Using Generalized q-Sampling Imaging

**DOI:** 10.1371/journal.pone.0133001

**Published:** 2015-07-13

**Authors:** Chao-Yu Shen, Yeu-Sheng Tyan, Li-Wei Kuo, Changwei W. Wu, Jun-Cheng Weng

**Affiliations:** 1 School of Medical Imaging and Radiological Sciences, Chung Shan Medical University, Taichung, Taiwan; 2 Department of Medical Imaging, Chung Shan Medical University Hospital, Taichung, Taiwan; 3 School of Medicine, Chung Shan Medical University, Taichung, Taiwan; 4 Division of Medical Engineering Research, National Health Research Institutes, Miaoli County, Taiwan; 5 Graduate Institute of Biomedical Engineering, National Central University, Taoyuan, Taiwan; National Yang-Ming University, TAIWAN

## Abstract

Radiation therapy is widely used for the treatment of brain tumors and may result in cellular, vascular and axonal injury and further behavioral deficits. The non-invasive longitudinal imaging assessment of brain injury caused by radiation therapy is important for determining patient prognoses. Several rodent studies have been performed using magnetic resonance imaging (MRI), but further studies in rabbits and large mammals with advanced magnetic resonance (MR) techniques are needed. Previously, we used diffusion tensor imaging (DTI) to evaluate radiation-induced rabbit brain injury. However, DTI is unable to resolve the complicated neural structure changes that are frequently observed during brain injury after radiation exposure. Generalized q-sampling imaging (GQI) is a more accurate and sophisticated diffusion MR approach that can extract additional information about the altered diffusion environments. Therefore, herein, a longitudinal study was performed that used GQI indices, including generalized fractional anisotropy (GFA), quantitative anisotropy (QA), and the isotropic value (ISO) of the orientation distribution function and DTI indices, including fractional anisotropy (FA) and mean diffusivity (MD) over a period of approximately half a year to observe long-term, radiation-induced changes in the different brain compartments of a rabbit model after a hemi-brain single dose (30 Gy) radiation exposure. We revealed that in the external capsule, the GFA right to left (R/L) ratio showed similar trends as the FA R/L ratio, but no clear trends in the remaining three brain compartments. Both the QA and ISO R/L ratios showed similar trends in the all four different compartments during the acute to early delayed post-irradiation phase, which could be explained and reflected the histopathological changes of the complicated dynamic interactions among astrogliosis, demyelination and vasogenic edema. We suggest that GQI is a promising non-invasive technique and as compared with DTI, it has better potential ability in detecting and monitoring the pathophysiological cascades in acute to early delayed radiation-induced brain injury by using clinical MR scanners.

## Introduction

Radiation therapy plays an important role in the treatment of both primary and metastatic brain tumors and can improve tumor control and overall survival in patients with inoperable or unresectable brain tumors. Besides, it can be used as adjuvant therapy after resection of high grade brain tumors. Inevitably, the exposure of normal brain tissues to radiation can lead to various side effects, such as cellular, axonal and vascular injury, and result in further neurological and behavioral deficits [1–3].

Generally, radiation-induced brain injury can be divided into three phases: acute phase (within 48 hours or during the first week), subacute to early delayed phase (1 week to 6 months) and late delayed phase (more than 6 months) [4–6]. Potential explanations for the histopathological changes have been provided by both glial and vascular hypotheses, which state that changes result from white matter demyelination with associated oligodendrocyte apoptosis, and also from coagulative and liquefactive tissue necrosis with associated vascular endothelial damage and hyalinization [7, 8]. However, neither the glial nor the vascular hypotheses can fully explain radiation-induced brain injury. A new hypothesis is that dynamic interactions between the multiple cell types, including vascular endothelial cells, oligodendrocytes, astrocytes, microglia, and neurons, are actively participating in the response to radiation-induced brain injury [3, 4, 6]. The pathophysiology of radiation-induced brain injury is not completely understood, and it remains a challenge for both basic scientists and clinical investigators.

Several studies with a number of animal models have demonstrated that in vivo magnetic resonance imaging (MRI) techniques, such as conventional T1- and T2-weighted imaging (T1WI and T2WI), magnetic resonance spectroscopy (MRS), diffusion-weighted imaging (DWI) and diffusion tensor imaging (DTI), are able to detect radiation-induced brain injury, thus revealing the importance of non-invasive imaging to assess brain damage caused by radiation therapy [5, 9–12].

Previously, we established a rabbit model for the longitudinal evaluation of radiation-induced brain injury using a 1.5 T clinical MR scanner with both T2WI and DTI indices. Multiple b-values, ranging incrementally from 0 to 2000 s/mm^2^ with a 250 s/mm^2^ interval, were used in that study [13]. We found that DTI indices could detect significant changes to the irradiated external capsule (white matter) but could not detect changes to the irradiated cerebral cortex, thalamus and hippocampus. These findings may help reveal the limitations of the DTI indices in detecting the predominance of relatively isotropic tissue and more complicated architecture containing substantial crossing or diverging fibers [14].

Recently, more advanced high angular resolution diffusion imaging (HARDI) models of the diffusion process, such as q-ball imaging (QBI), diffusion spectrum imaging (DSI), diffusion kurtosis imaging (DKI), neurite orientation dispersion and density imaging (NODDI), diffusion basis spectrum imaging (DBSI) and generalized q-sampling imaging (GQI), have been proposed to overcome the shortcomings of DTI for representing complex neural architecture. These techniques provide a more accurate, higher-order description of the water diffusion process than DTI provides [14–20].

To improve the evaluation of the neurotoxic adverse effects of irradiation treatment in both gray and white matter structures, we longitudinally evaluated the changes in various brain compartments with a clinical MR scanner using GQI indices mappings (same multiple b-values as our previous published DTI indices), including generalized fractional anisotropy (GFA), quantitative anisotropy (QA), and the isotropic value (ISO) of the orientation distribution function (ODF) on our previously established adult rabbit model. In addition, we compared GQI indices with DTI indices, including fractional anisotropy (FA) and mean diffusivity (MD) to detect single dose (30 Gy) cerebral hemisphere radiation-induced rabbit brain injury [13].

## Materials and Methods

This study presents a new GQI-based analysis of the exact same diffusion MRI and histopathology datasets that were described in our previous publication [13]. For completeness, we describe the previously-reported details of animal preparation, data acquisition, and histopathological evaluation below. Our MR image analysis methods are different from those described previously [13].

### Ethics statement

This study was carried out in strict accordance with the recommendations in the Guide for the Care and Use of Laboratory Animals of the Chung Shan Medical University. The protocol was approved by the Chung Shan Medical University animal ethics committee (permit number 919). In both irradiation and MRI scan, all animal procedures were performed under general anesthesia using an inhalation anesthesia mix of isoflurane and oxygen. In histopathological evaluation, subcutaneous Xylazine injection followed by muscular Tiletamine-Zolazepam injection was performed for deep anesthesia before perfusion fixation. All efforts were made to minimize suffering.

### Animal preparation

Five male, one-year-old New Zealand rabbits received irradiation of 30 Gy (collimations = 4 cm x 5 cm; source-skin distance (SSD), 98.5 cm) of the right hemisphere of the brain with a single highly collimated 6 MV photon beam from a Varian CL21EX linear accelerator (Varian, Palo Alto, CA, USA) under general anesthesia using an inhalation anesthesia mix of isoflurane (4% induction and 1.5% maintenance) and oxygen (400 mL/min) via a homemade plastic nasal/oral mask. The unirradiated left hemisphere of the brain served as an internal control for the subsequent analyses.

### Data acquisition

In our study, a brain MRI was performed on the rabbits before irradiation (baseline) and at the 1^st^, 2^nd^, 4^th^, 8^th^, 12^th^, 16^th^, 20^th^, 24^th^, 32^nd^, 40^th^ and 48^th^ weeks after irradiation (12 time points covering the acute to the chronic phases) using a 1.5 Tesla MR scanner (Magnetom Sonata, Siemens Medical Solutions, Erlangen, Germany) with double loop array coils.

Whole brain coronal T2WI images were acquired using a turbo spin echo with the following parameters: repetition time (TR) = 4330 ms, echo time (TE) = 114 ms, field of view (FOV) = 50 x 50 mm^2^, matrix size = 256 x 128, resolution (voxel size) = 0.195 x 0.39 x 1.5 mm^3^, number of excitations (NEX) = 13, number of slices = 30, and scan time = 9 min 33 sec.

Whole brain coronal multiple shells diffusion data were obtained using a multi-slice, single-shot spin echo EPI sequence with TR = 2900 ms, TE = 128 ms, FOV = 50 x 50 mm^2^, matrix size = 64 x 64 and resolution = 0.78 x 0.78 x 2 mm^3^. Twelve slices were acquired contiguously from the genu of the corpus callosum to the end of the cerebrum using 12 diffusion-encoding directions with b values from 0 to 2,000 s/mm^2^ (with a 250 s/mm^2^ interval), total number of diffusion encodings = 97 and the scan time was 42 min.

### MR Image analysis

**Fig 1:**
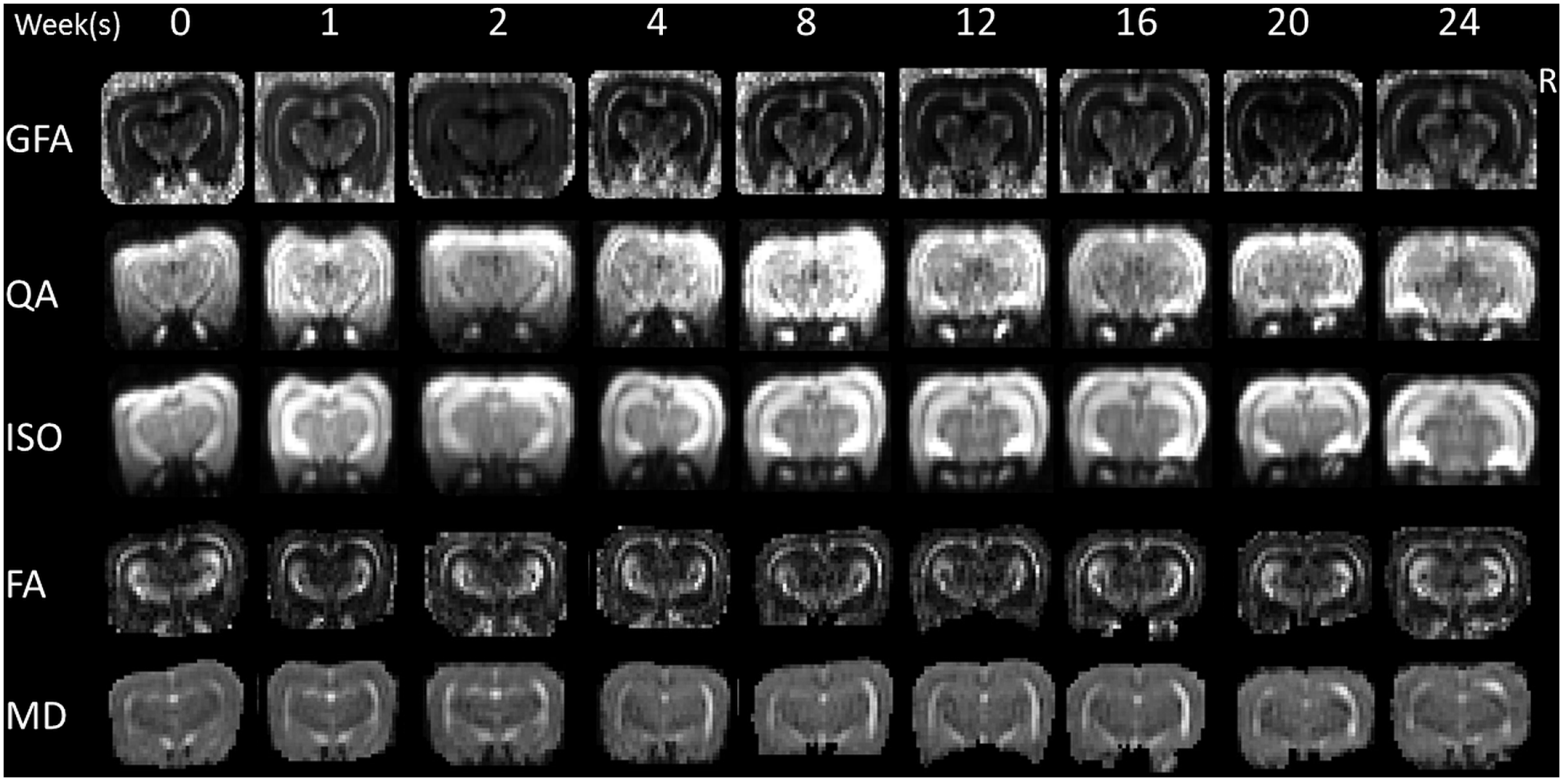
GFA, QA, ISO, FA and MD mappings. They were calculated from the multiple shells diffusion data with GQI and DTI methods, respectively, using DSI studio at the baseline (0) and at the 1^st^ to the 24^th^ weeks post-irradiation.

**Fig 2:**
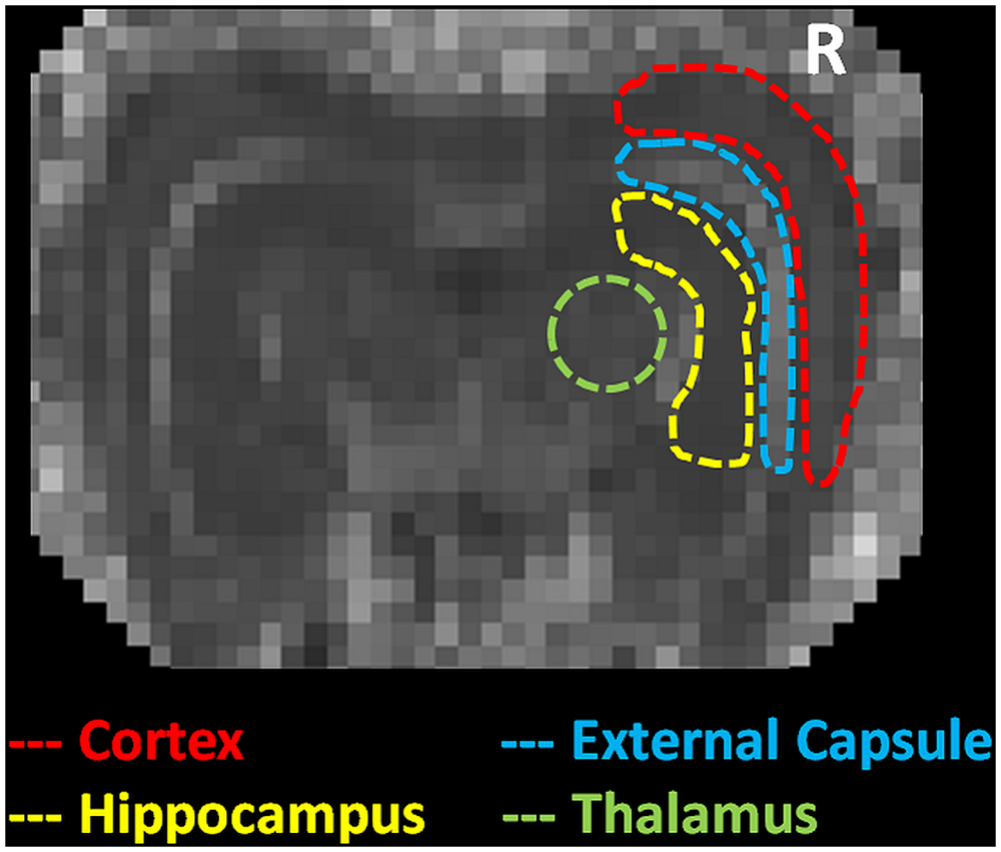
ROI drawings of four different compartments in one representative rabbit’s brain.

Before data analysis, denoising was performed using homemade software running in MATLAB [21]. After denoising, GQI indices mappings (including GFA, QA and ISO) and DTI indices mappings (including FA and MD) were calculated from multiple shells diffusion data using DSI studio (NTU, Taipei, Taiwan) (Fig 1). Using ImageJ (NIH, MD, USA), regions of interest (ROIs) were drawn manually on three consecutive slices of the GQI and the DTI indices mappings in four different compartments, including the bilateral cerebral cortex, external capsules, hippocampi and thalami (Fig 2). All results were expressed as the means ± standard error (SE), and the ratios of right (R, injury) to left (L, control) hemispheres were calculated for statistical analysis. Paired t-tests were used to detect significant differences between the pre- and the post-irradiation time points. A p-value < 0.05 was considered statistically significant.

### Histopathological evaluation

One experimental rabbit was sacrificed for histopathological evaluation after the 48^th^-week MRI scans. The whole brain was removed after the perfusion fixation of the rabbit with 4% paraformaldehyde, and coronal sections were prepared at four levels of the cerebrum. The cerebral tissues were embedded in paraffin, sectioned at 5 μm, and stained with hematoxylin and eosin (H&E). Luxol fast blue (LFB) staining was performed to detect myelin in the white matter.

## Results

### General appearance

We found progressive local hair loss on the right side of the head in all five experimental rabbits 2 weeks after irradiation. Two rabbits displayed an abnormal gait by the 24^th^ week and the 40^th^ week post-irradiation, and the other three rabbits showed no gross neurological abnormalities. Three rabbits died 26, 32 and 33 weeks after irradiation, possibly as a result of poor eating and drinking. Therefore, not all five rabbits completed the MRI studies at all following time points and only the MRI data from the baseline to the 24^th^ week were included for forward statistical analysis. After the 48^th^ week of MRI scans, one of the remaining two rabbits was sacrificed for histopathological examination.

### GQI indices analysis

**Fig 3:**
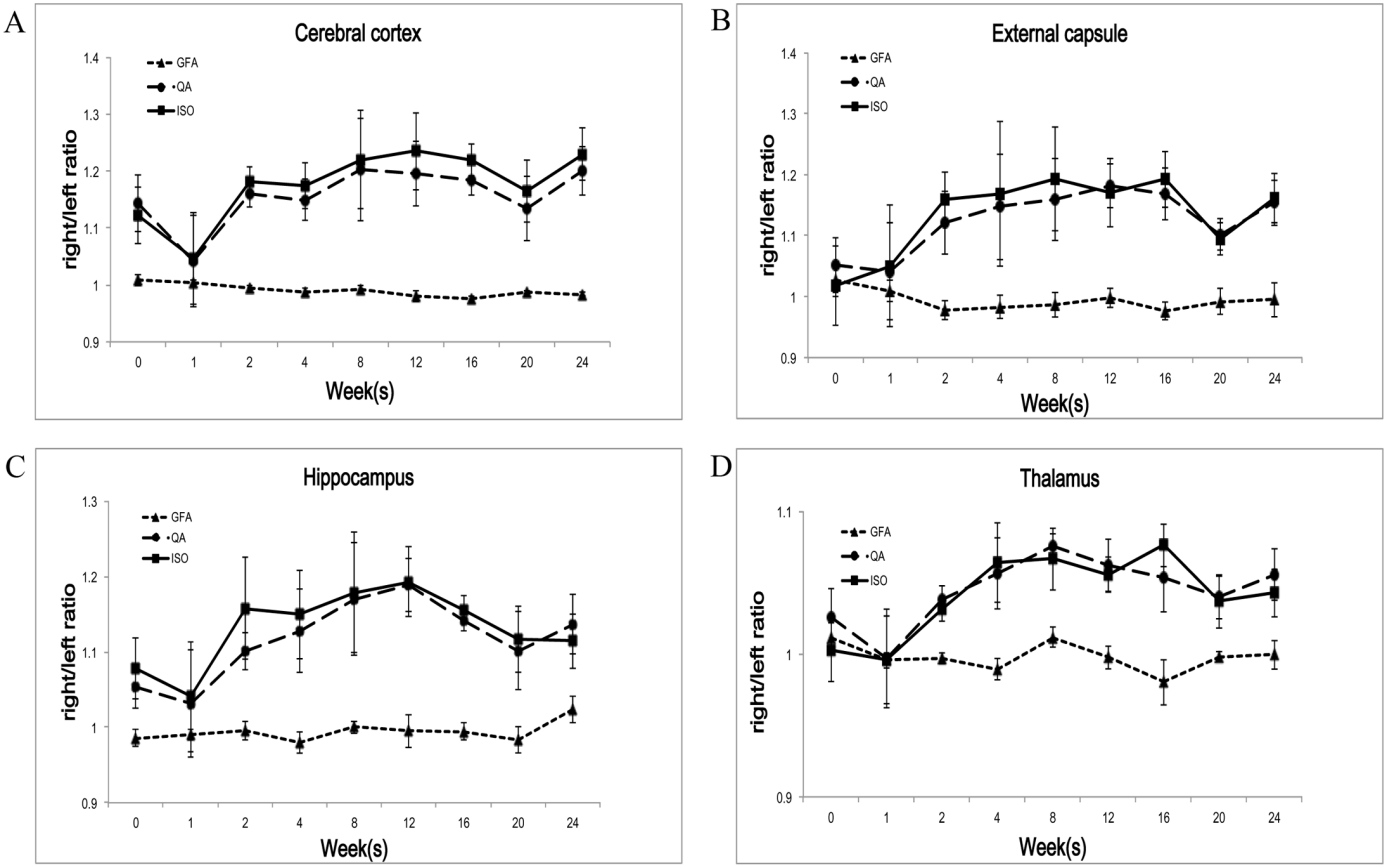
Longitudinal changes of the GQI indices in the different brain compartments after irradiation. (A) In the cerebral cortex, there was no clear trend in the GFA R/L ratio. The QA and ISO R/L ratios showed a rapid increase after the 1^st^ week followed by a plateau and then a gradual decrease. (B) In the external capsule, the GFA R/L ratio showed a gradual decrease during the initial 2 weeks followed by a gradual recovery. The QA and ISO ratios showed a rapid increase during the initial 2 weeks followed by a plateau and then a gradual decrease. (C) In the hippocampus, there was no clear trend in the GFA R/L ratio. The QA and ISO R/L ratios showed a rapid increase after the 1^st^ week followed by a plateau and then a gradual decrease. (D) In the thalamus, there was no clear trend in the GFA R/L ratio. The QA and ISO R/L ratios showed a rapid increase at the 1^st^ week followed by a plateau and then a gradual decrease.

In the cerebral cortex, the differences for the GFA R/L ratio reached statistical significance at the 4^th^, 12^th^, and 16^th^ weeks (p-value = 0.001, 0.002, 0.036) but there was no clear trend in the GFA R/L ratio. The QA and ISO R/L ratios showed a rapid increase after the 1^st^ week followed by a plateau and then a gradual decrease. The differences for the QA and ISO R/L ratios did not reach statistical significance at any of the following time points (Fig 3A and S1 Table).

In the external capsule, there was a gradual decrease during the initial 2 weeks followed by a gradual recovery in the GFA R/L ratio. The differences for the GFA R/L ratio reached statistical significance at the 16^th^ week (p = 0.031). The QA and ISO ratios showed a rapid increase during the initial 2 weeks followed by a plateau and then a gradual decrease. The differences for the QA and ISO R/L ratio did not reach statistical significance at any of the following time points (Fig 3B and S1 Table).

In the hippocampus, there was no clear trend in the GFA R/L ratio. The QA and ISO R/L ratios showed a rapid increase after the 1^st^ week followed by a plateau and then a gradual decrease. The differences for the QA R/L ratio reached statistical significance at the 12^th^ week (p = 0.014) and the 16^th^ week (p = 0.007) (Fig 3C and S1 Table).

In the thalamus, there was no clear trend in the GFA R/L ratio. The QA and ISO R/L ratios showed a rapid increase after the 1^st^ week followed by a plateau and then a gradual decrease. The differences for the ISO R/L ratio reached statistical significance at the 16^th^ week (p-value = 0.047) (Fig 3D and S1 Table).

**Fig 4:**
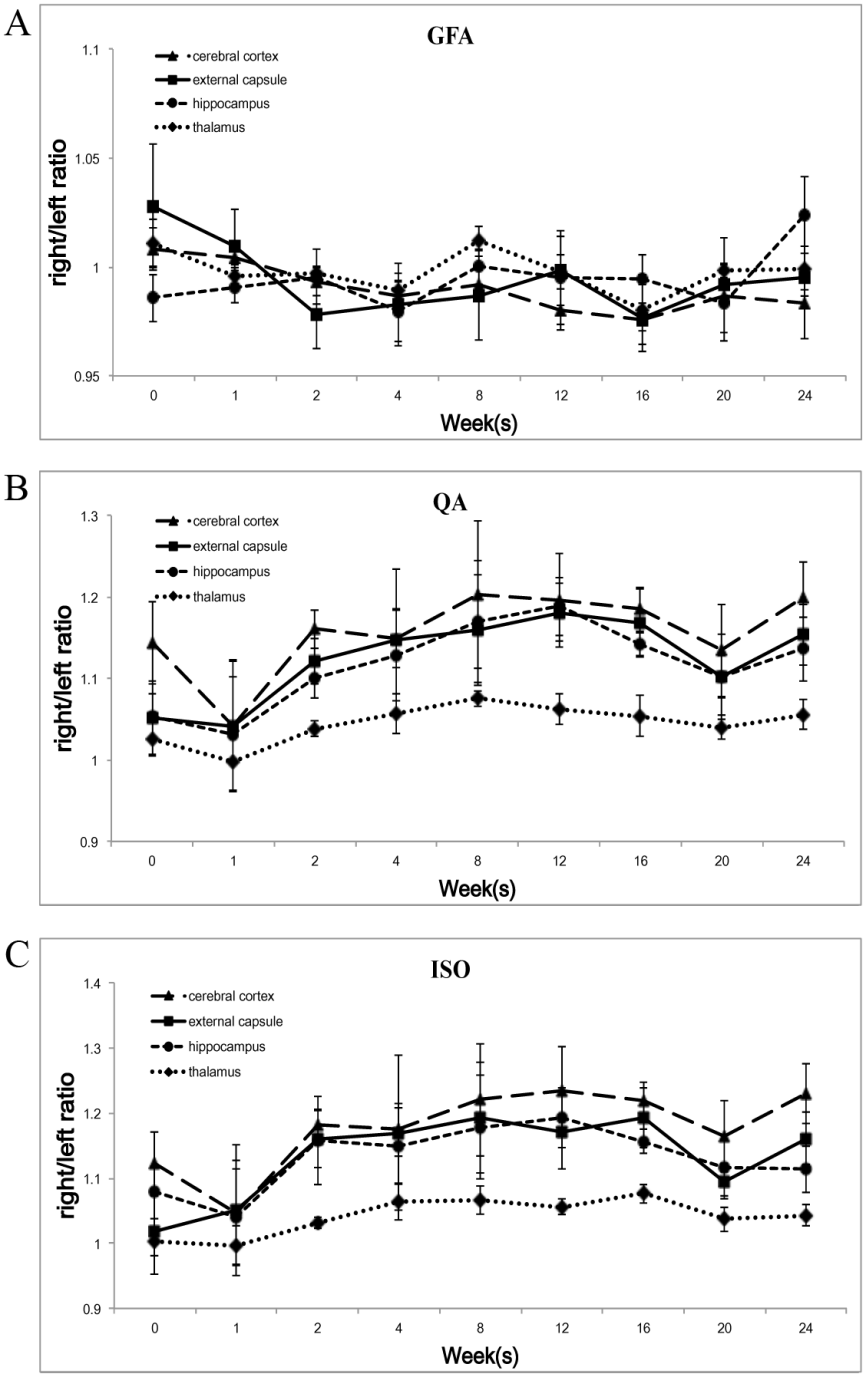
Longitudinal changes in the different brain compartments of the different GQI indices after irradiation. (A) The GFA R/L ratio showed a gradual decrease, followed by a gradual recovery in the external capsule, but no clear trends in the remaining three brain compartments. In both the QA and ISO R/L ratios, all four different brain compartments showed similar trends with a rapid increase after the 1^st^ week followed by a plateau and then a gradual decrease. (B, C) Both the trends of the QA and ISO R/L ratios at the thalamus showed a more gradual slope as compared with the other three brain compartments.

In summary, the GFA R/L ratio showed a gradual decrease, followed by a gradual recovery in the external capsule but no clear trends in the remaining three brain compartments (Fig 4A). Both the QA and ISO R/L ratios showed similar trends in the all four compartments with a rapid increase after the 1^st^ week followed by a plateau and then a gradual decrease. Both the trends of the QA and ISO R/L ratios at the thalamus showed a more gradual slope as compared with the other three brain compartments (Fig 4B and 4C).

### Comparison between GQI and DTI indices

**Fig 5:**
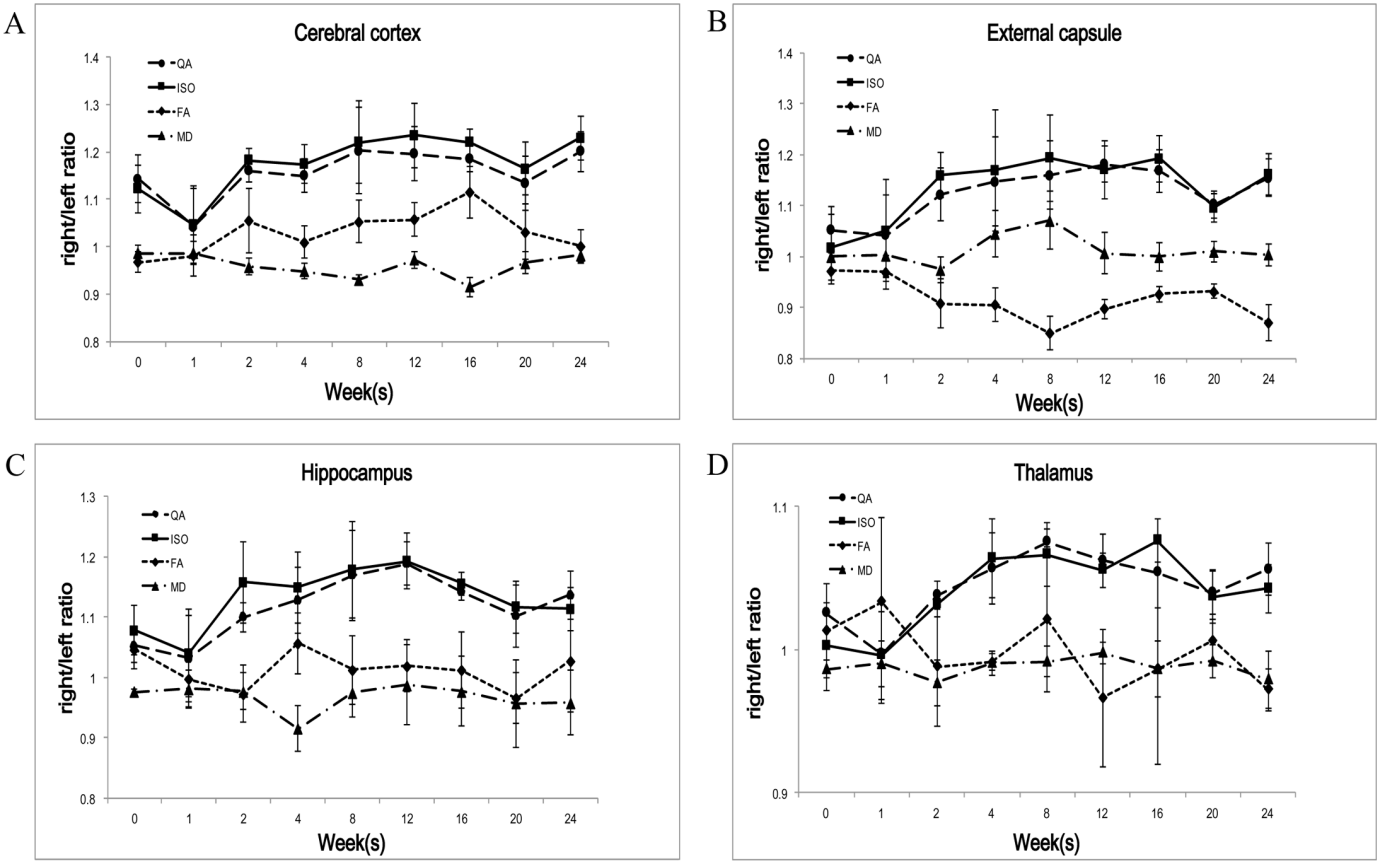
Longitudinal changes of the GQI indices and the DTI indices in the different brain compartments after irradiation. The GQI indices (QA and ISO) showed more clear trends compared with the DTI indices (FA and MD) in the all four compartments.

In the cerebral cortex, the difference for the MD R/L ratio reached statistical significance at the 8^th^ week (p-values = 0.025). There was no statistically significant change in the FA R/L ratio at any of the follow-up time points and no clear trend in both the FA and MD R/L ratios (Fig 5A and S1 Table).

In the external capsule, there was a continuing decrease followed by a gradual recovery in the FA R/L ratio, the differences reached statistical significance at the 8^th^ (p-value = 0.0193) and 12^th^ weeks (p-value = 0.037). There was no statistically significant change at any of the follow-up time points and no clear trend in the MD R/L ratio (Fig 5B and S1 Table).

In the hippocampus and thalamus, there was no statistically significant change at any of the follow-up time points and no clear trend in both the FA and MD R/L ratios (Fig 5C, 5D and S1 Table).

As compared with the DTI indices (FA and MD), the GQI indices (QA and ISO) showed more clear trends in the all four compartments and more statistically significant follow-up time points (Fig 5A–5D and S1 Table).

### Histopathology findings

**Fig 6:**
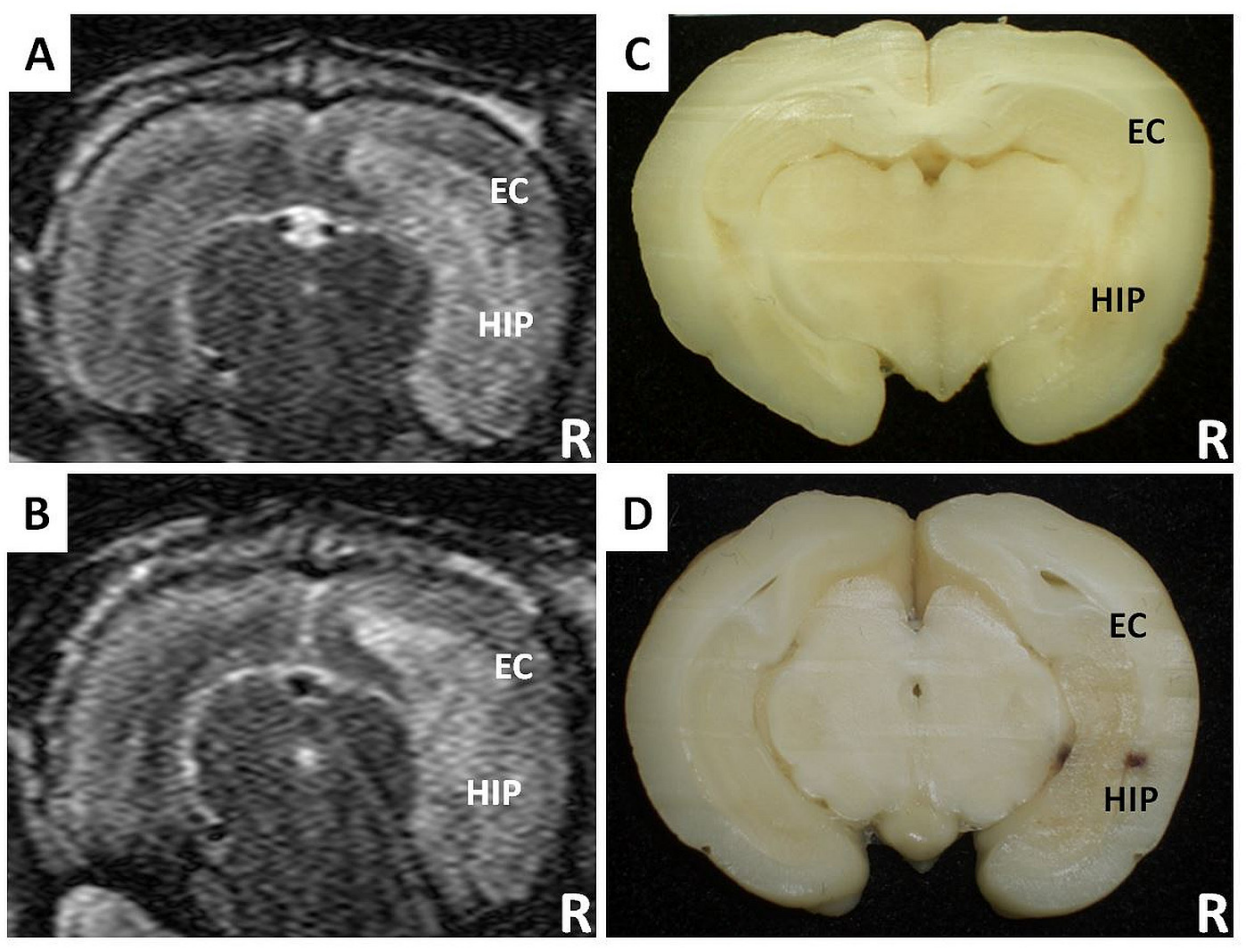
The change of signal intensity in the irradiated right hemisphere on T2 WI (A, B) correlated well with the gross morphology (C, D) of the sacrificed rabbit at the 48^th^ week post-irradiation. It showed mixed signal intensity with a loss of the normal architecture, which presented as radiation necrosis with focal hemorrhage and yellow discoloration in the irradiated right hemisphere (EC = external capsule; HIP = hippocampus; R = right side).

**Fig 7:**
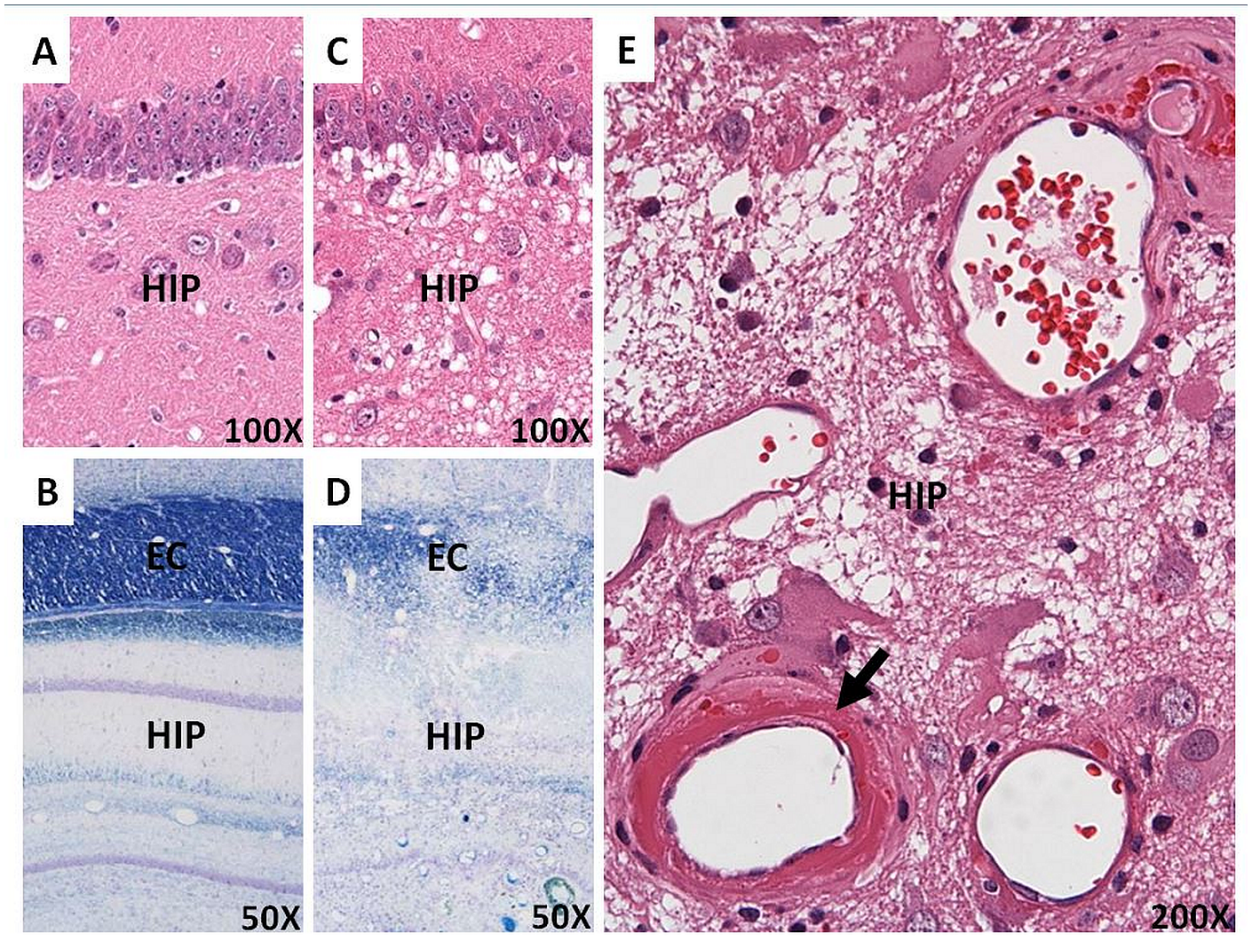
Histopathological evaluations of the injured (right) and control (left) sides of the rabbit brain at 48 weeks post-irradiation, including H&E (A, C, E) and LFB (B, D) staining. (A, B) No significant radiation-related alterations were observed in the left hemisphere of the brain. (C) Demyelination in the right hippocampus. Decreased coloration, indicating a loss of the myelin sheath in the right external capsule. (D) Disorganization of the myelin fibers in the right external capsule and right hippocampus. (E) Prominent hyalinization and fibrinoid deposition of the vessel walls (arrow) within the right hippocampus. (EC = external capsule; HIP = hippocampus).

One experimental rabbit was sacrificed for histopathological evaluation after the 48^th^ week of MRI scans. In the gross observation, we found yellow discoloration with focal hemorrhage in the right hemisphere but not in the left hemisphere (Fig 6). In the H&E sections, we found large areas of confluent coagulation necrosis with multifocal hemorrhagic foci involving the right external capsule and hippocampus. These areas were characterized by disorganized nerve tissue with amorphous cell debris, prominent fibrinoid necrosis of the vessel walls with wall thickening, and some luminal thrombosis (Fig 7B and 7E). In the LFB sections, the coloration in the right external capsule and hippocampus was decreased, indicating the loss of the myelin sheath. Disorganized myelin fibers were also observed (Fig 7D). In contrast, there were no significant gross morphological changes or histopathological alterations in the remaining right hemisphere (including the cerebral cortex and thalamus) or in the non-irradiated left hemisphere (Fig 7A and 7C).

## Discussion

Non-invasive DTI can distinguish cerebral microscopic structures, especially in the white matter regions. However, the microstructure of brain tissue changes upon brain injury, leading to greater non-Gaussian diffusion and non-monoexponential b-value dependence, and DTI is unable to resolve this complicated neural structure. In our previous study, we revealed that fractional anisotropy (FA), which representing the water diffusion anisotropy and indicating white matter tract integrity, showed a continuing decrease and followed by a gradual recovery in the external capsule (white matter) during acute to early delayed post-irradiation phase. However, in detecting relatively isotropic tissue and more complicated architectures such as cerebral cortex, thalamus and hippocampus, the DTI indices showed no significant change at any of the post-irradiation following time points [13]. Thus, approaches that are more accurate and sophisticated than conventional DTI are needed to extract additional information for these restricted diffusion environments. To overcome this limitation, many advanced diffusion imaging methods were recently developed for assessing white matter injury, such as QBI, DSI, DKI, NODDI, DBSI and GQI [14–20]. GQI describes the water diffusion behavior by the Fourier relationship between water diffusion and signal decay. The use of this relationship ensures more sensitive characterization than fitting the signal decay to a monoexponential model. We think GQI is not unique in its ability to detect pathological changes as compared to other HARDI methods, however, GQI is a unique q-space reconstruction method that can reconstruct ODF from a variety of diffusion datasets, including multiple-shell dataset used in this study, which can be obtained from most clinical scanners. As compared to the much known DTI which is limited to its Gaussian tensor model, GQI is believed to be able to provide more accurate directional and quantitative information about the complicated neural structures. A recent study compared GQI (with only one b-value of 1000 s/mm^2^) to DTI for the visualization of nerve fiber tracts within peritumoral edema of the brain and found that GQI tractography was superior to DTI for visualizing the tracts in the peritumoral edema of brain tumors. The authors found that the fiber tracts in edema displayed by DTI tractography were incomplete, missing, or ruptured and they suggested that GQI might help in the preoperative planning of surgical tumor resections [22]. In addition to GQI tractography, there are several indices derived from the GQI method, including GFA, QA and ISO. The GQI-derived diffusion indices are believed to be able to improve the level of significance and specificity in the analysis along the axial direction [17]. Our recent study used GQI and DTI indices for developing rabbit brain evaluation and found that both GOI and DTI indices were able to characterize the white matter anisotropy changes, whereas GQI provided further information about the gray matter area [23]. According to our results, we found that the GQI indices (QA and ISO) showed their advantages, with more clear trends and more statistically significant follow-up time points as compared with the DTI indices (FA and MD) on our adult rabbit model. To the best of our knowledge, no previous animal studies used a clinical MR scanner with GQI indices to evaluate multi-compartment longitudinal radiation-induced brain injury.

GFA is calculated from an ODF and it has high correlation with FA. In this study, we found that in the external capsule, the GFA R/L ratio showed similar trends as the FA R/L ratio of our previous study, but no clear trend was found in the remaining three brain compartments. The above findings could be explained by that the external capsule is a white matter structure, consisting of well-oriented fiber tracts with anisotropic diffusion environment. Therefore, the voxel-based GFA could show its ability in detecting the post-irradiated external capsule injury. However, the value of GFA decreased in detecting relatively isotropic tissue or more complicated architectures, such as cerebral cortex, thalamus and hippocampus. Besides, in the cortex, the GFA R/L ratio showed much smaller standard error as compared with the QA and ISO R/L ratios, which might enhanced the insensitivity of the GFA in detecting isotropic diffusion environment and resulting in more statistical significant time points even without clear trend. QA is calculated from the peak orientations on an ODF and it can be used to filter false fibers in a crossing fiber scenario. ISO is the minimum distribution value of an ODF, and thus it represents background isotropic diffusion. In a recent published study, the authors used two different phantoms for DTI index (FA) and GQI indices (GFA and QA) comparison. They found that QA was less sensitive to the partial volume effects of crossing fibers and free water, and the QA-aided tractography had better resolution than the FA-aided and the GFA-aided tractography [24].

The radiation-induced brain injuries in acute to early delayed phase are noted as transient, usually reversible and resolve spontaneously. The onset and duration of the injuries varies depending on factors such as radiation dose, radiation method, and the age at irradiation [5, 9, 25]. Post-irradiation reactive astrogliosis, which is characterized by astrocytic proliferation and acts as diffusion barriers [26], and may resulting in increased QA but decreased ISO. In a previous rat model study with histopathological conformation, the authors found an initially increase, followed by a gradual reduction of reactive astrogliosis in longitudinal post irradiation brain injury evaluation [5]. Post-irradiation transient demyelination, which is characterized by impairment of myelin sheath and leads to reduced water diffusion restriction [27–29], and may resulting in decreased QA but increased ISO. Post-irradiation vasogenic edema is an early, readily-recognizable pathophysiological event. It is characterized by disruption of the blood-brain barrier (BBB) [30] and leads to increasing water diffusivity and may resulting in decreased QA but increased ISO. However, QA and ISO do not just reflect the result of any single mechanism, such as reactive astrogliosis, demyelination or vasogenic edema. Indeed, they reflect the results of the above mentioned complicated dynamic interactions. In our study, both the QA and ISO R/L ratios showed similar trends in the all four different compartments with a rapid increase after the 1^st^ week post-irradiation, followed by a plateau and then a gradual decrease. We found that the effects of reactive astroglosis, demyelination and vasogenic edema may not be obvious in the post-irradiation acute phase, and resulting in initially no clear trend of either the QA or ISO R/L ratio in the all four different compartments. But, once the effects of reactive astrogliosis, demyelination and vasogenic edema showed their advantage, the QA and ISO R/L ratios rapidly increased. The increase of the QA could be majorly caused by the result of increased reactive astroglosis and the increase of the ISO could be explained by the result of increased demyelination and vasogenic edema. The ISO R/L ratios showed relative more sensitive to detect post-irradiation brain change with a steeper slope as compared with the QA R/L ratios (Fig 3), it could be explained by that both demyelination and vasogenic edema lead to increased water diffusivity and background isotropic diffusion. Furthermore, both the QA and ISO R/L ratios showed a gradual decrease after the 16^th^ week post-irradiation, which corresponded with that post-irradiation acute to early delayed brain injury is transient and usually resolve spontaneously [31].

The histopathological examination confirmed that a single-dose exposure was successfully delivered to only the right hemisphere of the brains of rabbits. However, the permanent demyelination and vascular injury were limited to the exposed external capsule and hippocampus but not found in the cerebral cortex or thalamus. The results suggested that the late delayed radiation-induced brain injuries are predominantly characterized by white matter injury and the hippocampus, which contains neurogenic neurons and nerve fibers, is relatively more radio-sensitive than the cerebral cortex and thalamus which are made up of mostly non-dividing neurons. These irreversible changes are the main contributor to the morbidity and mortality of radiation-induced brain injury [7].

There are several limitations in our study. First, we used a relatively small number of experimental rabbits, resulting in a lack of adequate histopathological comparisons with MR images at each post-irradiation following time point. Although there are relatively complete histopathological studies in the literature that provided us with sufficient information for an MRI-histopathology correlation, we agree that a histopathological correlation at each time point can improve the impact of the results and should be considered in further study. Second, according to previous studies [13, 25, 32, 33], 30 Gy seemed feasible for longitudinal evaluation of radiation-induced brain injury in rabbit model, but actually, three of the five (60%) experimental rabbits had subsequently died 26, 32 and 33 weeks after irradiation. Therefore, decreasing radiation dose may be taken into consideration for further longitudinal evaluation of radiation-induced brain injury in rabbit model, especially to evaluate late delayed effects (more than 6 months). Third, our results showed that in some of the pre-radiation baseline GQI and DTI indices were higher or lower in right side than in left side (R/L ratio above or below 1), which may result from magnetic field inhomogeneity or other cause of artifacts and may be considered as a drawback of the hemi-brain radiation design of irradiated/unirradiated comparison, but unlike single lateral analysis, this method could diminish interscan variation and it had worked well in many previous animal MRI studies [5, 9–11]. Fourth, we did not perform neurobehavioral and cognitive evaluations for clinical and MR images comparison. In a recent study, the authors successfully established a rabbit model reproducing the functional and neurostructural consequences of near-term intrauterine growth restriction [34]. The study used both the Open Field Behavioral Test and the Object Recognition Task for neurobehavioral and cognitive evaluation and found good correlation between the DTI indices and neurobehavioral and cognitive outcomes. This valuable result may help improving further study design for post-irradiation rabbit brain injury models by using a comprehensive comparison, including MR images, clinical neurobehavioral and cognitive and histopathological evaluations. Last, the 1.5 T clinical MR scanner provided relatively poor image resolution and demanded more time than the high-field MRI scanner, which could result in decreased sensitivity for lesion detection but may shorten the “distance” from the bench to the bedside.

## Conclusions

Our results showed the GQI indices, especially the QA and ISO may reflect the post-irradiation complicated dynamic interactions in the rabbit brain. We suggest that GQI is a promising non-invasive technique and as compared with DTI, it has better potential ability to detect and monitor the pathophysiological cascades in acute to early delayed radiation-induced brain injury in both gray matter and white matter structures by using clinical MR scanners.

## Supporting Information

S1 Checklist NC3Rs The ARRIVE Guidelines Checklist. (PDF)

S1 Table Evaluating GQI and DTI indices (R/L ratio) from baseline to post-irradiation 24 weeks in the four different rabbit brain compartments. (DOC)
