# Supplementary material for: Quantitative Evaluation of Rabbit Brain Injury after Cerebral Hemisphere Radiation Exposure Using Generalized q-Sampling Imaging
Source: PLoS One. 2015 Jul 13;10(7):e0133001. doi: 10.1371/journal.pone.0133001 (PMC4500591; doi:10.1371/journal.pone.0133001)
Supplement: S1 Table — (DOC) [file pone.0133001.s002.doc]

**S1 Table. Evaluating GQI and DTI indices (R/L ratio) from baseline to post-irradiation 24 weeks in the four different rabbit brain compartments**

|  | | **0w** | **1w** | **2w** | **4w** | **8w** | **12w** | **16w** | **20w** | **24w** |
| --- | --- | --- | --- | --- | --- | --- | --- | --- | --- | --- |
| **Cortex R/L** | |  |  |  |  |  |  |  |  |  |
| **GFA** | **Mean** | 1.0083 | 1.0045 | 0.9933 | *0.9869 | 0.9919 | *0.9804 | *0.9757 | 0.9867 | 0.9837 |
|  | **SE** | 0.0097 | 0.0048 | 0.0065 | 0.0064 | 0.0062 | 0.0093 | 0.0051 | 0.0026 | 0.0030 |
| **QA** | **Mean** | 1.1431 | 1.0418 | 1.1605 | 1.1493 | 1.2024 | 1.1952 | 1.1847 | 1.1347 | 1.2003 |
|  | **SE** | 0.0504 | 0.0803 | 0.0235 | 0.0359 | 0.0904 | 0.0574 | 0.0271 | 0.0569 | 0.0427 |
| **ISO** | **Mean** | 1.1221 | 1.0465 | 1.1814 | 1.1744 | 1.2203 | 1.2348 | 1.2198 | 1.1646 | 1.2294 |
|  | **SE** | 0.0498 | 0.0811 | 0.0252 | 0.0406 | 0.0861 | 0.0679 | 0.0281 | 0.0553 | 0.0455 |
| **FA** | **Mean** | 0.9669 | 0.9812 | 1.0541 | 1.0100 | 1.0534 | 1.0573 | 1.1154 | 1.0317 | 1.0018 |
|  | **SE** | 0.0219 | 0.0432 | 0.0684 | 0.0353 | 0.0448 | 0.0358 | 0.0544 | 0.0582 | 0.0333 |
| **MD** | **Mean** | 0.0219 | 0.0432 | 0.0684 | 0.0353 | *0.0448 | 0.0358 | 0.0544 | 0.0582 | 0.0333 |
|  | **SE** | 0.0167 | 0.0242 | 0.0182 | 0.0162 | 0.0102 | 0.0168 | 0.0212 | 0.0238 | 0.0151 |
| **External Capsule R/L** | |  |  |  |  |  |  |  |  |  |
| **GFA** | **Mean** | 1.0278 | 1.0092 | 0.9784 | 0.9828 | 0.9869 | 0.9983 | *0.9764 | 0.9918 | 0.9950 |
|  | **SE** | 0.0284 | 0.0172 | 0.0161 | 0.0190 | 0.0206 | 0.0159 | 0.0153 | 0.0219 | 0.0280 |
| **QA** | **Mean** | 1.0518 | 1.0411 | 1.1214 | 1.1472 | 1.1592 | 1.1811 | 1.1680 | 1.1019 | 1.1540 |
|  | **SE** | 0.0450 | 0.0799 | 0.0281 | 0.0877 | 0.0679 | 0.0351 | 0.0418 | 0.0255 | 0.0376 |
| **ISO** | **Mean** | 1.0177 | 1.0506 | 1.1595 | 1.1690 | 1.1934 | 1.1705 | 1.1920 | 1.0954 | 1.1610 |
|  | **SE** | 0.0655 | 0.1000 | 0.0440 | 0.1190 | 0.0854 | 0.0563 | 0.0455 | 0.0270 | 0.0400 |
| **FA** | **Mean** | 0.9716 | 0.9695 | 0.9073 | 0.9053 | *0.8487 | *0.8966 | 0.9256 | 0.9315 | 0.8697 |
|  | **SE** | 0.0260 | 0.0329 | 0.0485 | 0.0321 | 0.0328 | 0.0185 | 0.0146 | 0.0134 | 0.0354 |
| **MD** | **Mean** | 0.9994 | 1.0026 | 0.9748 | 1.0458 | 1.0706 | 1.0063 | 0.9995 | 1.0094 | 1.0029 |
|  | **SE** | 0.0257 | 0.0436 | 0.0254 | 0.0457 | 0.0574 | 0.0410 | 0.0277 | 0.0200 | 0.0227 |
| **Hippocampus R/L** | |  |  |  |  |  |  |  |  |  |
| **GFA** | **Mean** | 0.9857 | 0.9908 | 0.9954 | 0.9798 | 1.0001 | 0.9950 | 0.9943 | 0.9835 | 1.0237 |
|  | **SE** | 0.0109 | 0.0073 | 0.0125 | 0.0143 | 0.0081 | 0.0217 | 0.0111 | 0.0175 | 0.0176 |
| **QA** | **Mean** | 1.0527 | 1.0315 | 1.1004 | 1.1280 | 1.1697 | *1.1886 | *1.1417 | 1.1017 | 1.1364 |
|  | **SE** | 0.0280 | 0.0708 | 0.0243 | 0.0553 | 0.0747 | 0.0358 | 0.0139 | 0.0520 | 0.0394 |
| **ISO** | **Mean** | 1.0783 | 1.0405 | 1.1575 | 1.1493 | 1.1786 | 1.1928 | 1.1560 | 1.1166 | 1.1140 |
|  | **SE** | 0.0406 | 0.0731 | 0.0680 | 0.0584 | 0.0802 | 0.0459 | 0.0188 | 0.0437 | 0.0359 |
| **FA** | **Mean** | 1.0461 | 0.9973 | 0.9732 | 1.0565 | 1.0131 | 1.0194 | 1.0115 | 0.9659 | 1.0271 |
|  | **SE** | 0.0322 | 0.0455 | 0.0480 | 0.0498 | 0.0565 | 0.0437 | 0.0629 | 0.0412 | 0.0846 |
| **MD** | **Mean** | 0.9765 | 0.9809 | 0.9768 | 0.9153 | 0.9755 | 0.9881 | 0.9774 | 0.9574 | 0.9586 |
|  | **SE** | 0.0048 | 0.0310 | 0.0303 | 0.0381 | 0.0411 | 0.0653 | 0.0570 | 0.0725 | 0.0527 |
| **Thalamus R/L** | |  |  |  |  |  |  |  |  |  |
| **GFA** | **Mean** | 1.0111 | 0.9957 | 0.9968 | 0.9895 | 1.0118 | 0.9975 | 0.9804 | 0.9982 | 0.9994 |
|  | **SE** | 0.0108 | 0.0054 | 0.0036 | 0.0075 | 0.0069 | 0.0079 | 0.0159 | 0.0034 | 0.0099 |
| **QA** | **Mean** | 1.0256 | 0.9970 | 1.0380 | 1.0566 | 1.0753 | 1.0623 | 1.0539 | 1.0397 | 1.0559 |
|  | **SE** | 0.0207 | 0.0346 | 0.0097 | 0.0250 | 0.0094 | 0.0186 | 0.0248 | 0.0149 | 0.0182 |
| **ISO** | **Mean** | 1.0029 | 0.9961 | 1.0454 | 1.0638 | 1.0665 | 1.0556 | *1.0761 | 1.0370 | 1.0427 |
|  | **SE** | 0.0221 | 0.0308 | 0.0163 | 0.0279 | 0.0218 | 0.0122 | 0.0151 | 0.0188 | 0.0166 |
| **FA** | **Mean** | 1.0130 | 1.0332 | 0.9880 | 0.9914 | 1.0209 | 0.9663 | 0.9866 | 1.0065 | 0.9721 |
|  | **SE** | 0.0200 | 0.0594 | 0.0411 | 0.0059 | 0.0507 | 0.0479 | 0.0664 | 0.0151 | 0.0145 |
| **MD** | **Mean** | 0.9862 | 0.9903 | 0.9769 | 0.9907 | 0.9916 | 0.9977 | 0.9867 | 0.9921 | 0.9794 |
|  | **SE** | 0.0151 | 0.0159 | 0.0162 | 0.0085 | 0.0107 | 0.0074 | 0.0195 | 0.0114 | 0.0200 |

* P < 0.05
